# ASO Author Reflections: Evaluating the Effectiveness of Axillary Reverse Mapping in Reducing Breast Cancer-Related Lymphedema Among Ethnically Diverse Patients

**DOI:** 10.1245/s10434-024-15663-5

**Published:** 2024-07-04

**Authors:** Fardeen Bhimani, Sheldon Feldman, Maureen McEvoy

**Affiliations:** Breast Surgery Division, Department of Surgery, Montefiore Medical Center, Montefiore Einstein Comprehensive Cancer Care, Bronx, NY USA

## Past

Historically, breast cancer-related lymphedema (BCRL) has been a significant and debilitating complication after surgery. Previous studies have documented that minority populations face a twofold increased risk of developing lymphedema compared with their nonminority counterparts,^1–3^ highlighting a crucial disparity in healthcare outcomes. This increased risk of BCRL has been attributed to various factors, including difficulties in access to care, comorbid conditions, and race and ethnicity. Addressing these disparities is vital for improving outcomes in breast cancer treatment. Our study evaluated the effectiveness of Axillary Reverse Mapping (ARM) combined with Bioimpedance Spectroscopy (BIS) in reducing the incidence of BCRL within an ethnically diverse patient cohort.^4^ Specifically, we analyzed whether ARM could provide a significant clinical advantage and reduce the higher risk of lymphedema that is traditionally seen in minority populations.

## Present

Our study’s findings are particularly noteworthy given our cohort’s demographic composition; 83% of participants belong to ethnic minority groups. Previous studies have documented a higher obesity rate in African-American patients, thus predisposing them to a higher rate of BCRL. Furthermore, studies have shown that African-American and Hispanic patients have a higher odds of developing BCRL. Contrary to previous findings, our study participants, despite having a mean body mass index (BMI) of 29.5+5.7 kg/m^2^, 34% being African-American, and 45.3% identifying as Hispanic, our BCRL rate remained at 1.4%. Moreover, our study found that combining Axillary Reverse Mapping (ARM) with Bioimpedance Spectroscopy (BIS) for early detection and intervention resulted in a significantly lower incidence of BCRL. Our overall lymphedema rate was 1.4%, with SLNB contributing 0% and Axillary Lymph Node Dissection (ALND) contributing 8.3% of the total. These rates are significantly lower than previous estimates of 5–7% for SLNB and 25–30% for ALND. These findings highlight the effectiveness of ARM in lowering lymphedema rates, especially in high-risk populations. Furthermore, the incorporation of BIS enabled the early detection and management of lymphedema, preventing progression to more severe stages and improving patient quality of life. This proactive approach not only improves clinical outcomes but also serves as a model for reducing healthcare disparities in breast cancer treatment.

## Future

To build on these promising findings, future research should include larger, multicenter studies that validate our findings across a wide range of healthcare settings and populations. Expanding the sample size, particularly among patients undergoing ALND, is critical for confirming ARM's efficacy. Additionally, we look forward to the results of the Alliance A221702: Axillary Reverse Mapping, which is a prospective phase 3 trial designed to study rates of lymphedema and regional recurrence after sentinel lymph node biopsy and sentinel lymph node biopsy followed by axillary lymph node dissection, with and without axillary reverse mapping (ARM). This trial will provide further insights into the effectiveness and applicability of ARM. Moreover, cost-effectiveness analyses will provide useful information about the economic benefits of implementing ARM as a standard practice. Long-term follow-up studies are needed to fully understand the long-term effects of ARM on lymphedema incidence and its effect on patient's quality of life. Finally, it is essential to include diverse patient populations in research to ensure that advances in surgical oncology benefit everyone equally. By prioritizing diversity, we can reduce disparities in lymphedema outcomes and promote equitable health care for all patients.
